# A comparison of four different approaches to measuring health utility in depressed patients

**DOI:** 10.1186/1477-7525-11-81

**Published:** 2013-05-09

**Authors:** Nicholas Turner, John Campbell, Tim J Peters, Nicola Wiles, Sandra Hollinghurst

**Affiliations:** 1 Centre for Mental Health, Addiction and Suicide Research, School of Social and Community Medicine, University of Bristol, Oakfield House, Canynge Hall, 39 Whatley Road, Bristol BS8 2PS, UK; 2 Primary Care Research Group, Peninsula Medical School, Smeall Building, St Luke’s Campus, Magdalen Road, Exeter EX1 2LU, UK; 3 School of Clinical Sciences, University of Bristol, 69 St Michael’s Hill, Bristol BS2 8DZ, UK; 4 Centre for Academic Primary Care, School of Social and Community Medicine, University of Bristol, Canynge Hall, 39 Whatley Road, Bristol BS8 2PS, UK

**Keywords:** Depression, EQ-5D, SF-6D, Health related utility, QALYs

## Abstract

**Background:**

A variety of instruments are used to measure health related quality of life. Few data exist on the performance and agreement of different instruments in a depressed population. The aim of this study was to investigate agreement between, and suitability of, the EQ-5D-3L, EQ-5D Visual Analogue Scale (EQ-5D VAS), SF-6D and SF-12 new algorithm for measuring health utility in depressed patients.

**Methods:**

The intraclass correlation coefficient (ICC) and Bland and Altman approaches were used to assess agreement. Instrument sensitivity was analysed by: (1) plotting utility scores for the instruments against one another; (2) correlating utility scores and depressive symptoms (Beck Depression Inventory (BDI)); and (3) using Tukey’s procedure. Receiver Operating Characteristic (ROC) analysis assessed instrument responsiveness to change. Acceptability was assessed by comparing instrument completion rates.

**Results:**

The overall ICC was 0.57. Bland and Altman plots showed wide limits of agreement for each pair wise comparison, except between the SF-6D and SF-12 new algorithm. Plots of utility scores displayed ’ceiling effects’ in the EQ-5D-3L index and ’floor effects’ in the SF-6D and SF-12 new algorithm. All instruments showed a negative monotonic relationship with BDI, but the EQ-5D-3L index and EQ-5D VAS could not differentiate between depression severity sub-groups. The SF-based instruments were better able to detect changes in health state over time. There was no difference in completion rates of the four instruments.

**Conclusions:**

There was a lack of agreement between utility scores generated by the different instruments. According to the criteria of sensitivity, responsiveness and acceptability that we applied, the SF-6D and SF-12 may be more suitable for the measurement of health related utility in a depressed population than the EQ-5D-3L, which is the instrument currently recommended by NICE.

## Background

The National Institute for Health and Care Excellence (NICE) guidance on the management of depression identifies several priorities including accurate case recognition, optimal use of medication and effective delivery of psychological therapies [1]. This guidance highlights the need to identify cost-effective interventions and the use of cost per quality adjusted life years (QALYs) for this purpose. Measuring health related quality of life (HRQoL) to construct QALYs can be conducted in a number of ways, including using various ‘off the shelf’ instruments to define and value an individual’s health state (known as health utility) [2]. However, if the scores produced by different instruments differ markedly, this will impact on estimates of cost-effectiveness that are obtained and may lead to discrepant or uncertain conclusions as to whether or not an intervention should be recommended/funded.

NICE currently recommends using the EQ-5D-3L questionnaire to measure HRQoL, though this instrument has been widely criticised for a number of reasons for example, being insensitive in certain conditions such as depression [3,4] and for ignoring considerable individual variation in the ordering of health states [5]. Research comparing methods of calculating HRQoL has previously shown that different instruments do not provide comparable estimates of HRQoL. For example, Raisch *et al.*, 2012 found poor levels of agreement between the SF-6D, Health Utilities Index 2, Health Utilities Index 3 and a Feelings Thermometer within a Diabetic population [6]. This lack of comparability has led some observers to suggest that in certain conditions one instrument may be recommended above others. For example, the Health Utilties Index 3 has recently been recommended as the most suitable instrument for measuring QALYs in studies of retinopathy [7]. The accurate measurement of HRQoL in depressed patients is particularly important given the impact this condition has on physical, emotional and social aspects of an individual’s overall well-being [8]. However, the investigation of health utility in patients with depression has been very limited, despite depression being a leading cause of disability worldwide [9]. The vast majority of previous comparison studies have not included samples from this population, have focused on common mental health disorders grouped together (not just depression) or used a very small sample size, presented mainly summary statistics or only assessed one aspect of instrument suitability [10-12]. As such the comparability of values of HRQoL produced by different instruments in patients with depression is unclear. The evidence from other disease areas suggests that the instruments are unlikely to provide comparable scores. It is therefore important to investigate this and, if true, to determine whether one instrument may be more suitable than others. Furthermore, the development of a relatively new approach to calculating health utility based on use of the SF-12 questionnaire highlights the need to understand the differences between the various instruments used to calculate health utility [13].

The aim of the current study was to investigate the extent of agreement between, and the suitability of, different instruments for measuring health utility in depressed patients. Data from the Cognitive behavioural therapy as an adjunct to pharmacotherapy for treatment resistant depression in primary care (CoBalT) trial [14] were used to compare the health utility values obtained from four different instruments: (1) EQ-5D-3L [15,16]; (2) EQ-5D Visual Analogue Scale (EQ-5D VAS) [17]; (3), SF-6D [18]; and (4) SF-12 new algorithm [13], for primary care based patients with treatment resistant depression. The aspects investigated were: the level of agreement between the health utility values generated by the instruments; instrument sensitivity (ceiling/floor effects and discriminatory ability); responsiveness of the instruments to changes in depressed state; and the acceptability of the instruments in terms of completion rates. We assessed whether the four approaches to measuring health utility in depressed patients could be used interchangeably, and if not, which method performed best in this setting.

## Methods

### Participants

This was a secondary analysis of data collected as part of the CoBalT study, a randomised controlled trial examining the effectiveness of cognitive behavioural therapy as an adjunct to usual care that included pharmacotherapy for patients with treatment resistant depression in primary care. Individuals were eligible for the trial if they were aged between 18–75 years, were currently taking antidepressant medication and had been doing so at an adequate dose for at least 6 weeks, scored 14 or more on the Beck Depression Inventory (BDI) (second version) [19] and met the ICD-10 criteria for depression (assessed using the Clinical Interview Schedule – Revised form [20]). Participants were followed up at intervals of three months for a year with health utility information being collected at baseline, six and twelve months [14].

### Health utility measures

Health utility data were collected from self-completed questionnaires that participants completed in the presence of a research assistant who could provide clarification about what a question was asking, if required.

#### EQ-5D-3L

The EQ-5D-3L provides a simple, generic, single index value reflecting HRQoL. It comprises five self-report items that ask about five health domains with respect to “today” with three possible response categories: 1) no problems; 2) some problems; and 3) severe problems. The EQ-5D-3L is therefore able to represent 243 (3^5^) distinct health states [15]. These states may then be converted into a single index value by applying published reference weights (for CoBalT, the UK population valuation set [16]) to each domain response and subtracting the total of these weights from one [2].

#### EQ-5D visual analogue scale

The EQ-5D VAS also seeks to assign individuals a single index value representing health status. Respondents are asked to record how good or bad their health is on a vertical thermometer-like line with a scale ranging from zero (representing worst imaginable state) to 100 (best imaginable state). The reported figure may then be rescaled by dividing by 100 to scale between zero and one [17].

#### SF-6D

The SF-6D algorithm provides a way of estimating a preference-based single index measure from a generic quality of life questionnaire (the Short-Form 36 (SF-36)) [18], that is a common outcome in many health research studies including randomised trials. Health states can be derived from the six multi-level health dimensions of the SF-36 questionnaire. In total, 18,000 different health states can be described: two dimensions have six levels, three dimensions have five levels and one dimension has four levels (6^2^ × 5^3^ × 4^1^ = 18,000). Preference weights for each dimension allow the prediction of health utility values for all possible health states [18]. The briefer 12-item (SF-12 revised acute version) quality of life questionnaire [21] was used in the CoBalT study. At the time the CoBalT study began, no algorithm was available to permit the calculation of health utility values from the SF-12. Therefore, an additional four questions from the SF-36 were included, in line with established procedures [2], to allow the SF-6D algorithm to be applied to derive health utility scores for each individual.

#### SF-12 New algorithm instrument

The SF-12 questionnaire is commonly used as a HRQoL outcome measure. It contains 12 items that map onto 12 of the 36 items from the SF-36 questionnaire. Brazier & Roberts’ have recently published an algorithm [13] that permits estimation of a preference based measure from the SF-12 questionnaire (without the need for additional questions as detailed for the SF-6D algorithm).

### Depression measure

Depression was measured using the BDI, which is a self-report inventory consisting of 21 multiple choice questions about the respondent’s feelings in the past two weeks. Each question has four potential response options that score from 0–3. The scores for each question can be summed to produce a total score ranging from 0 to 63. This total score can then be categorised according to severity: (1) not depressed (BDI score <14); (2) mild depression (BDI score 14–19); (3) moderate depression (BDI score 20–28) and (4) severe depression (BDI score ≥29) [19].

### Statistical analysis

All analyses were carried out using Stata (version 12.1). The assessments of agreement, sensitivity and acceptability were carried out using the 12 month follow up data. Analysis of responsiveness to change was carried out comparing the 12 month data with baseline (as this was that comparison made in the economic evaluation of the CoBalT trial).

### Agreement

To avoid differences in estimates of cost-effectiveness arising from the use of varying instruments, the four instruments should generate health utility scores that have a high level of agreement. The intraclass correlation coefficient (ICC) was calculated to assess the overall agreement between the four different methods for calculating health utility. Pairwise agreements between the different instrument scores (such as EQ-5D-3L index vs. SF-6D, EQ-5D-3L index vs. SF-12 new algorithm) was assessed using the Bland and Altman approach [22]. For each pairwise analysis the difference between the two measures was plotted against the mean measurement for those two instruments for each individual, along with the limits of agreement (the range of values that would be expected to include 95% of individual differences).

### Sensitivity

A good instrument should be able to produce scores for various degrees of ill and good health with an adequate degree of accuracy, effectively detecting and representing differences between individuals. Previous studies have reported that certain instruments may lack sensitivity at the tails of the utility index [23,24]. Potential ‘ceiling’ and ‘floor’ effects were examined by plotting the health utility scores generated by each of the different instruments against one another.

To be a useful and valid measure of health utility in depressed patients the health utility scores generated by the instruments should decrease monotonically with worsening depression score and should differ markedly between groups based on severity of depression [25]. These hypotheses were investigated by calculating the Spearman’s rank correlation coefficients between the health utility scores and BDI scores, and by carrying out the Tukey-Kramer multiple comparison procedure testing for differences between groups based on severity of depression [26,27].

### Responsiveness to change

An instrument measuring health utility should be able to detect and represent a change in an individual’s health over time. A binary variable was created dichotomising participants into those with at least a 50% reduction in BDI score at 12 months compared to baseline and those who did not (the primary outcome in the CoBalT trial), and a variable representing the change in health utility score in this time period was also generated. Receiver Operating Characteristic (ROC) curves (a plot of sensitivity versus 1-specificity) for the instruments were then plotted. The area under an ROC curve (AUC) is a measure of the discriminatory ability of an instrument as it represents how accurately the change in health utility score reflects whether or not an individual is classified as improved or not [28,29]. The equality of the AUCs produced for the instruments was then tested using the method of De Long et al. [30].

### Acceptability

Acceptability was assessed with respect to the completion rates of the four instruments [31,32]. The proportion of respondents who provided enough information for the calculation of a health utility score for each of the different instruments was calculated and compared by means of the Marascuilo Procedure [33,34]. If the absolute difference between two proportions is greater than the calculated critical value this suggests that there is evidence of a difference between the completion rates.

## Results

Of the 469 individuals who were randomised within the CoBalT study, 396 were followed up at 12 months. A health utility score could be calculated for 395 participants using the EQ-5D-3L, for 394 using the EQ-5D VAS and for 393 using the SF-6D and the SF-12 new algorithm. A total of 393 participants had completed all four instruments. The mean scores for the four instruments are similar; however, there was greater variance associated with the EQ-5D based measures (Table 1).

**Table 1:** Descriptive statistics at baseline and 12 month follow-up

**Characteristic**	**EQ-5D-3L**	**EQ-5D VAS**	**SF-6D**	**SF-12 new algorithm**
	**(n=395)**	**(n=394)**	**(n=393)**	**(n=393)**
**Age**				
*mean (SD)*	50.0 (11.6)	50.0 (11.6)	49.9 (11.6)	49.9 (11.6)
**Gender**				
Female *n (%)*	293 (74.2%)	293 (74.4%)	291 (74.1%)	291 (74.1%)
Male *n (%)*	102 (25.8%)	101 (25.6%)	102 (25.9%)	102 (25.9%)
**Suffered from depression in the past**				
Yes	353 (89.4%)	352 (89.3%)	351 (89.3%)	351 (89.3%)
No	42 (10.6%)	42 (10.7%)	42 (10.7%)	42 (10.7%)
**Duration of current episode of depression**				
<2 years	95 (24.1%)	95 (24.1%)	94 (23.9%)	94 (23.9%)
1-2 years	70 (17.7%)	70 (17.8%)	70 (17.8%)	70 (17.8%)
>2 years	230 (58.2%)	229 (58.1%)	229 (58.3%)	229 (58.3%)
**BDI score at baseline**				
*mean (SD)*	31.3 (10.4)	31.3 (10.4)	31.2 (10.4)	31.2 (10.4)
**BDI score at 12 months**				
*mean (SD)*	19.3 (13.6)	19.3 (13.7)	19.2 (13.5)	19.2 (13.5)
Health utility score at baseline*				
*mean (SD)*	0.55 (0.31)	0.51 (0.20)	0.56 (0.11)	0.57 (0.08)
**Health utility score at 12 months**				
*mean (SD)*	0.60 (0.35)	0.59 (0.25)	0.62 (0.16)	0.62 (0.14)

*n=390 for the SF-6D and SF-12 new algorithm for this variable.

### Agreement

**Figure 1:**
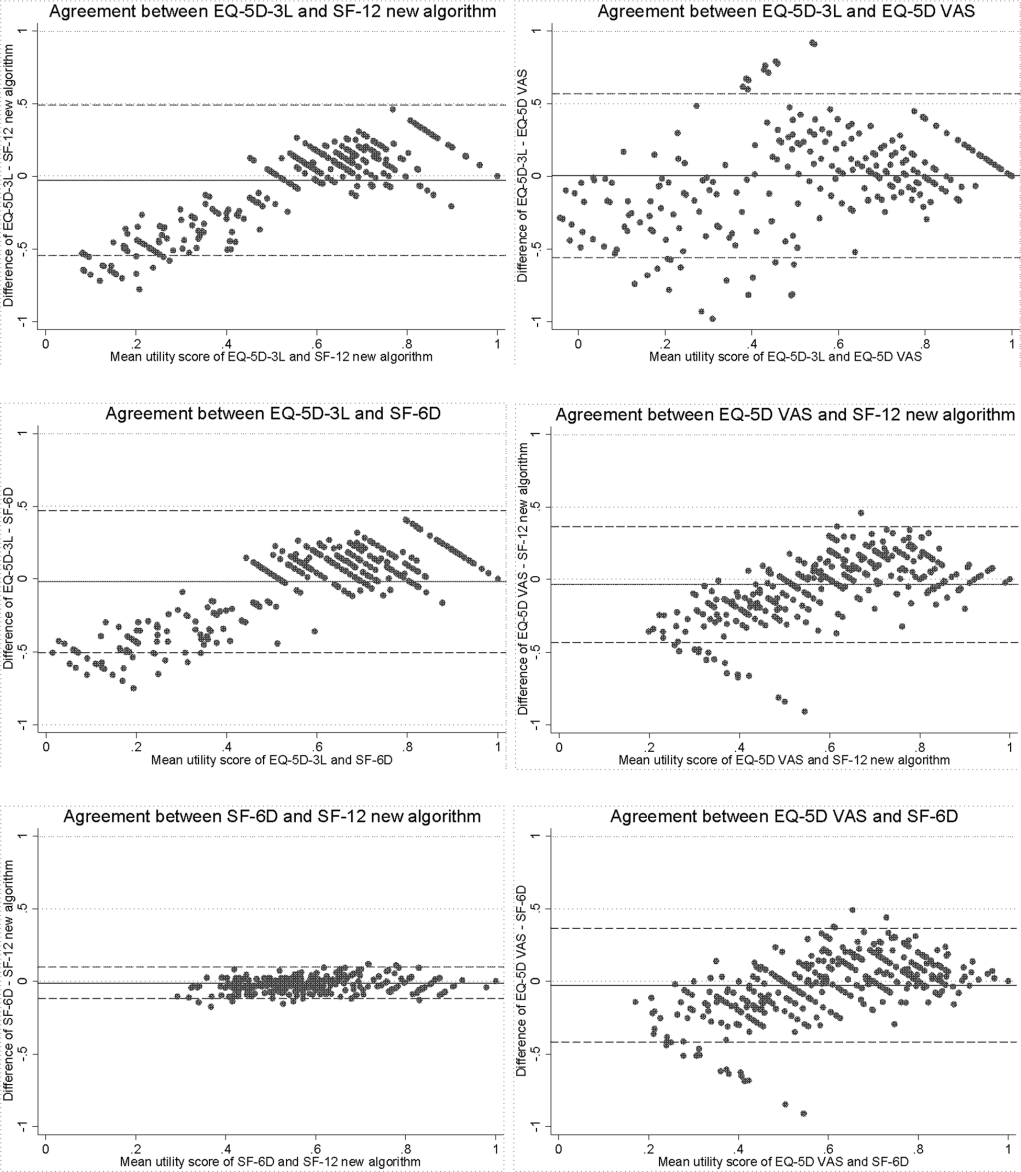
Bland and Altman plots for agreement of health utility scores between instruments.

The overall ICC was 0.57, suggesting a fairly low level of agreement between the four instruments. This was reinforced by the mixed-effects repeated measures ANOVA procedure used to calculate the ICC, which provided evidence of an instrument effect (p=0.01). The Bland and Altman plots for each pair-wise comparison showed wide limits of agreement, except for the comparison between the SF-6D and SF-12 new algorithm, suggesting these were the only two instruments that could be used reasonably interchangeably (Figure 1). The plots also showed that there was systematic variation between the scores produced by the EQ-5D based instruments (EQ-5D-3L and EQ-5D VAS) and the SF-6D and SF-12 new algorithm. Less healthy individuals (those with a utility score of <0.5) tended to have a higher score on the SF-6D and SF-12 new algorithm compared with the EQ-5D based instruments, whereas healthier individuals tended to have relatively high scores on the EQ-5D based instruments.

### Sensitivity

**Figure 2:**
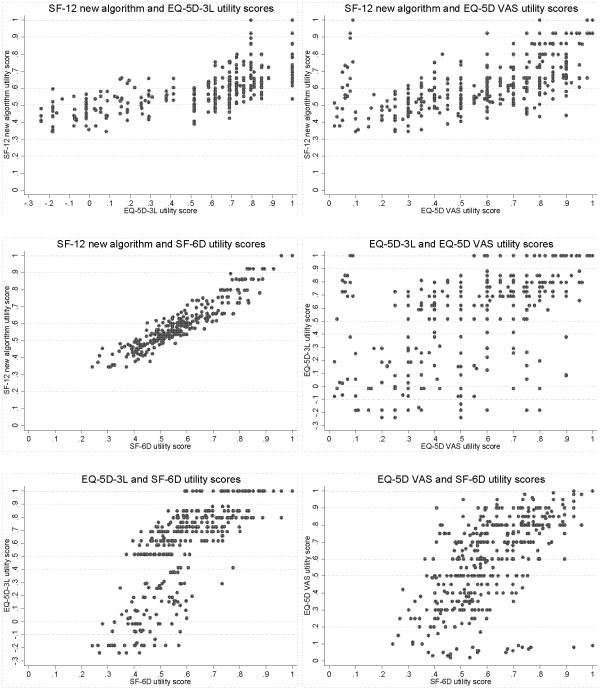
Scatter plots of health utility scores displaying ceiling and floor effects of instruments.

Plots of the health utility score generated by each of the different instruments against one another showed evidence of ceiling effects in the EQ-5D-3L index scores and floor effects in the SF-6D and SF-12 new algorithm (Figure 2). When the EQ-5D-3L index shows “full health” (a utility score of 1) the corresponding scores on the SF-6D and SF-12 new algorithm were wide-ranging. The plots also display a gap with no observations for the EQ-5D-3L index between scores of 0.833 and 1; this represents a range in which it is not possible to score using this instrument [32]. At the opposite end of the scale, however, when the EQ-5D-3L index shows a score of 0 or below (representing a state “equivalent to” or “worse than death”) the scores on the SF-6D and SF-12 new algorithm were, at their lowest, only 0.241 and 0.345 respectively. Plots with the EQ-5D VAS showed a large amount of variation between scores.

The SF-12 new algorithm had the strongest negative monotonic relationship with BDI score (Spearman’s rho = −0.715). The other instruments also showed negative monotonic relationships with BDI score but of smaller magnitude: Spearman’s rho SF-6D = −0.689; EQ-5D-3L index = −0.628; EQ-5D VAS = −0.529. The Tukey-Kramer multiple comparison procedure provided evidence that health utility scores generated by the EQ-5D based instruments did not differ between mild and moderate depression severity groups (Table 2). The SF-6D and SF-12 new algorithm instruments produced utility scores that differed between depression severity groups.

**Table 2:** Ability of instruments to discriminate between levels of depression severity

**Depression severity groups**	**Utility score means by depression severity group *mean (SD)***		**Mean difference in health utility score**	**Tukey-Kramer test statistic (critical value = 3.649)^**
**EQ-5D-3L:**				
Not depressed vs Mild depression	0.797 (0.226)	0.670 (0.243)	0.128	4.242*
Mild depression vs Moderate depression	0.670 (0.243)	0.557 (0.295)	0.112	3.272
Moderate depression vs Severe depression	0.557 (0.295)	0.268 (0.352)	0.289	9.641*
**EQ-5D VAS:**				
Not depressed vs Mild depression	0.713 (0.239)	0.586 (0.229)	0.127	5.247*
Mild depression vs Moderate depression	0.586 (0.229)	0.561 (0.209)	0.025	0.901
Moderate depression vs Severe depression	0.561 (0.209)	0.422 (0.211)	0.139	5.693*
**SF-6D:**				
Not depressed vs Mild depression	0.725 (0.137)	0.624 (0.119)	0.102	7.843*
Mild depression vs Moderate depression	0.624 (0.119)	0.554 (0.096)	0.070	4.733*
Moderate depression vs Severe depression	0.554 (0.096)	0.482 (0.107)	0.072	5.505*
**SF-12 new algorithm:**				
Not depressed vs Mild depression	0.732 (0.139)	0.617 (0.096)	0.116	9.796*
Mild depression vs Moderate depression	0.617 (0.096)	0.564 (0.073)	0.053	3.927*
Moderate depression vs Severe depression	0.564 (0.073)	0.505 (0.083)	0.059	4.947*

^*The critical values for each comparison were the same at three decimal places.*

* Indicates a test statistic greater than the critical value from the Studentized range distribution at the 5% alpha level.

### Responsiveness to change

There was evidence of a difference in the ability of the instruments to reflect a change in participants’ levels of health. All instruments showed a reasonable ability to discriminate between individuals who had “improved” in terms of their BDI score and those who had not. The AUC of the SF-6D and SF-12 new algorithm were very similar and greater than those of the EQ-5D-3L and EQ-5D VAS. The test for equality yielded strong evidence that the AUCs were different from one another (p<0.0001) (Table 3). A sensitivity analysis was carried out varying the point at which the outcome was dichotomised. This had little impact on the results (data not shown).

**Table 3:** Responsiveness of instruments to improvement in depression

**Utility instrument**	**Area under the ROC curve**	**95% confidence interval**
EQ-5D-3L	0.71	0.66 – 0.76
EQ-5D VAS	0.68	0.63 – 0.74
SF-6D	0.81	0.76 – 0.85
SF-12 new algorithm	0.80	0.76 – 0.85

p-value<0.0001 for test of equality of the AUCs.

### Acceptability

The completion rates for each of the instruments were all very high and extremely similar: EQ-5D-3L = 99.7%; EQ-5D VAS = 99.5%; SF-6D = 99.2%; SF-12 new algorithm = 99.2%. The results of the Marascuilo Procedure provided no evidence for any differences between the proportions of respondents who provided enough information for the calculation of a health utility score for each of the four different instruments (data not shown).

## Discussion

### Findings

There was a substantial lack of agreement between health utility measures in depressed subjects. The size of the limits of agreement in the Bland and Altman plots suggest that only the SF-6D and SF-12 new algorithm instruments may be used relatively interchangeably. This lack of agreement highlights the importance and relevance of the second study objective (namely, assessing the suitability of the instruments within a population of depressed subjects) to those involved in health technology assessments and policymakers.

The findings suggest that the SF-6D and SF-12 new algorithm instruments had a greater sensitivity than the EQ-5D instruments in depressed subjects. As expected, all of the instruments showed a decreasing health utility score with increasing BDI score (with the SF-12 new algorithm having the strongest monotonic relationship), but when respondents were classified into subgroups according to severity of depression the EQ-5D based instruments could not adequately differentiate between those with mild and moderate depression. The better performance of the SF-6D and SF-12 new algorithm may be because there are a greater number of possible response categories, and hence health states, for the SF-12 questions compared with the EQ-5D-3L (18,000 and 243 respectively) [34,35].

The results of plotting instrument scores against one another also showed that the EQ-5D-3L was less sensitive than the SF-6D and SF-12 new algorithm instruments at the upper end of utility scores. There was a wide range of scores on the SF-6D and SF-12 new algorithm for those who scored one on the EQ-5D-3L index. This is likely to be because it is not possible to produce an index score for the EQ-5D-3L within the range 0.883 to 1, and again that there is a wider range of health states defined for the SF-6D and SF-12 new algorithm compared with the EQ-5D-3L [35,36]. At the other end of the scale, however, the SF-6D and SF-12 new algorithm instruments do not appear to be able to describe a large range of very poor health states. The formulae used to generate health utility scores from the SF-6D and SF-12 new algorithm do not produce values close to or below zero, and as such may be insensitive at the lower end of the scoring continuum [13,23]. The consequences of these ceiling and floor effects are that the EQ-5D-3L may not adequately differentiate between different health states at the top of the scale, whilst the SF-6D and SF-12 new algorithm instruments may not adequately differentiate between health states at the lower end of the scale. In terms of assessing changes in quality of life for specific interventions, this means that the SF-6D and SF-12 new algorithm instruments may underestimate changes in quality of life for individuals in poorer health, but are likely to be better than the EQ-5D-3L at detecting changes in those at the higher end of the scale and therefore at distinguishing between different severities of depression.

The SF-6D and SF-12 new algorithm instruments were better able to discriminate between those who had shown an improvement in depression severity and those who had not in ROC analysis. This may again be attributable to the greater number of response categories for the SF questionnaires. The time frame covered by the questions may also have impacted on instrument responsiveness; the EQ-5D-3L asks questions about health “today” whereas the acute version of the SF-12 questionnaire used in the CoBalT trial asks questions relating to “the past week”, which may mean that the latter is more sensitive to changes in health state compared with the EQ-5D-3L [23].

### Strengths and limitations

This investigation utilised a large sample size that was very similar for all of the instruments compared. As a secondary analysis of data the outcomes used in the investigation would have been approached by the study participants from a neutral point of view. However, no assessment of test-retest reliability for the four instruments measuring health utility was planned as part of trial.

Responsiveness to change was examined using ROC analysis, requiring the indicator of change to be a binary variable, which may sacrifice information relating to the size of change in health state [29]. On the other hand, a sensitivity analysis was carried out varying the point at which the outcome was dichotomised and this had no impact on the conclusions drawn.

Acceptability was assessed in terms of the completion rates of the four different instruments. Individuals completed the questionnaires in the presence of a researcher who was able to clarify the meaning of a question if required. It is probably therefore not surprising that completion rates for all four instruments were very high. It should therefore be noted that response rates might have been different, and differed more between instruments, had the instruments been completed without the presence of a research assistant – for example, as part of a postal or online questionnaire.

The participants in this study had treatment resistant depression and did not include those with a first or new episode of depression. However, it is widely recognised that many patients do not respond to antidepressants [37] and as such we believe our results to be generalisable.

### Context of findings within previous work

The measurement of health related utility in patients with depression has been very limited and the comparison of different instruments within this patient population is almost non-existent. Lamers et al. [10] compared the utility scores produced by the EQ-5D-3L and SF-6D and found evidence of a difference in the mean utility scores of the instruments. However, the patients recruited had a wide range of common mental health disorders, not just depression, and agreement was assessed by comparing the overall mean scores of the instruments [10]. To the best of our knowledge, only two previous studies have compared utility scores generated by the EQ-5D-3L and SF-6D in a sample of depressed individuals in primary care (one from the UK and one a sample from the Netherlands) [11,12]. The previous UK study suggested that the EQ-5D-3L and SF-6D performed similarly and that both were fairly insensitive to differences in severity of depression. However, this was a small study (*n=114*), presented primarily summary statistics and again only assessed agreement by comparison of the overall mean utility scores of the instruments [11]. The study from the Netherlands (using the Dutch EQ-5D tariff) only assessed responsiveness to change, and found little difference between the EQ-5D-3L and the SF-6D in a sample of 267 participants [12]. The current study adds to previous work by investigating not only the EQ-5D-3L and SF-6D but also including the EQ-5D VAS and SF-12 new algorithm, carrying out a more rigorous statistical analysis of agreement, sensitivity, responsiveness and acceptability and using a larger UK sample of depressed individuals.

## Conclusions

The lack of agreement between instruments measuring health utility in those with depression means that cost-effectiveness analyses may produce differing and potentially conflicting conclusions as to whether or not an intervention should be recommended for use in this population. In order for there to be consistency between the conclusions drawn from cost-effectiveness analyses a single instrument should be recommended and utilised in depression research – or, at the very least, there needs to be greater knowledge than is presently available about the implications of the choice of instrument. NICE currently recommends the EQ-5D-3L, but we found that the EQ-5D-3L was insensitive in depressed patients. The EQ-5D VAS is designed to elicit a participant’s own valuation of their HRQoL, rather than generating a population-based valuation. In the economic evaluation of medical interventions it is public money that is being considered and as such it seems appropriate to use population valuations. For this reason the EQ-5D VAS is unlikely to be used in cost-effectiveness analyses. There was no difference in the acceptability of the instruments in terms of completion rates, although, as outlined earlier, these completion rates may be artificially inflated by the presence of a research assistant who was able to clarify queries during questionnaire completion. The SF-6D and SF-12 new algorithm instruments outperformed the EQ-5D-3L (and EQ-5D VAS) in terms of instrument sensitivity and responsiveness to change. This suggests that the SF-6D and SF-12 new algorithm instruments may be more appropriate for use in depressed individuals and could be used relatively interchangeably. However, as the SF-12 is frequently included as an outcome measure in trials, the SF-12 new algorithm has the advantage that it permits evaluation of health utility without the need for an additional questionnaire, thus reducing patient burden and potentially increasing follow-up rates.

NICE guidance currently states that the EQ-5D-3L should be used for the estimation of QALYs in the assessment of medical interventions. However, NICE permits the use of alternative measures when there is evidence that the EQ-5D-3L is not appropriate within a specific patient group in terms of certain criteria such as validity and responsiveness [4]. A new version of the EQ-5D, the EQ-5D-5L, consisting of dimensions with five levels rather than three has been developed in an attempt to increase sensitivity and reduce the issue of ceiling effects. The valuation exercise for this instrument is still ongoing but once complete this instrument warrants further investigation of the type presented here [38]. Nevertheless, this study suggests that a depressed population may represent one such patient group where the current form of the EQ-5D is not the most suitable instrument for the measurement of health utility and an alternative method should be used. Further research is needed to confirm and contextualise these findings in populations of depressed patients who are not involved in a clinical trial setting, and where account may be taken of a range of data collection processes. In the meantime, we would recommend the use of the SF-12 based instruments for the assessment of HRQoL in depressed patients.

## Competing interests

All authors declare no conflict of interest.

## Authors’ contributions

NW and SH conceptualized the project and oversaw the analysis which was carried out by NT. All authors contributed to the drafting of the manuscript, read and approved the final manuscript.
